# Increasing dietary linoleic acid does not increase tissue arachidonic acid content in adults consuming Western-type diets: a systematic review

**DOI:** 10.1186/1743-7075-8-36

**Published:** 2011-06-10

**Authors:** Brian S Rett, Jay Whelan

**Affiliations:** 1 Department of Nutrition, University of Tennessee, Knoxville, Tennessee 37996-1900, USA

## Abstract

**Background:**

Linoleic acid, with a DRI of 12-17 g/d, is the most highly consumed polyunsaturated fatty acid in the Western diet and is found in virtually all commonly consumed foods. The concern with dietary linoleic acid, being the metabolic precursor of arachidonic acid, is its consumption may enrich tissues with arachidonic acid and contribute to chronic and overproduction of bioactive eicosanoids. However, no systematic review of human trials regarding linoleic acid consumption and subsequent changes in tissue levels of arachidonic acid has been undertaken.

**Objective:**

In this study, we reviewed the human literature that reported changes in dietary linoleic acid and its subsequent impact on changing tissue arachidonic acid in erythrocytes and plasma/serum phospholipids.

**Design:**

We identified, reviewed, and evaluated all peer-reviewed published literature presenting data outlining changes in dietary linoleic acid in adult human clinical trials that reported changes in phospholipid fatty acid composition (specifically arachidonic acid) in plasma/serum and erythrocytes within the parameters of our inclusion/exclusion criteria.

**Results:**

Decreasing dietary linoleic acid by up to 90% was not significantly correlated with changes in arachidonic acid levels in the phospholipid pool of plasma/serum (*p = 0.39*). Similarly, when dietary linoleic acid levels were increased up to six fold, no significant correlations with arachidonic acid levels were observed (*p = 0.72)*. However, there was a positive relationship between dietary gamma-linolenic acid and dietary arachidonic acid on changes in arachidonic levels in plasma/serum phospholipids.

**Conclusions:**

Our results do not support the concept that modifying current intakes of dietary linoleic acid has an effect on changing levels of arachidonic acid in plasma/serum or erythrocytes in adults consuming Western-type diets.

## Background

Arachidonic acid (AA, 20:4 n-6) is a potent bioactive molecule. When released from membrane phospholipids, it is converted to a variety of bioactive compounds, called eicosanoids. These oxidized lipid molecules are related to a number of chronic diseases including cardiovascular disease, cancer and inflammation [1-4]. Enrichment of AA in tissues is positively correlated with the production of eicosanoids. Linoleic acid (LA, 18:2 n-6) is the major dietary polyunsaturated fatty acid (PUFA) in the Western diet and is a metabolic precursor to AA, linked biochemically via two desaturases and an elongase. Typical intakes of LA are 12-17 grams per day for women and men, respectively [5], or approximately 6% of energy. In the absence of other omega-6 (n-6) PUFA (including dietary AA), dietary LA is the sole contributor to tissue AA. This relationship had been established in experimental rodent models where dietary LA was correlated with tissue AA content in a non-linear relationship in rats provided fat-free background diets [6] and lipid-rich diets [7].

Recent reviews suggest this relationship may exist in adult humans consuming a typical Western-type diet [8,9] and some have recommended limiting LA intake as a way to help reduce tissue AA levels [10,11]. Certainly, this relationship had been reported in subjects consuming diets containing LA at levels less than 2% of energy [12]. There are, however, a number of recent papers suggesting that increasing dietary LA does not increase tissue AA levels, but in fact may have an inverse relationship [13,14]. To compound the complexity of this relationship, the family of n-6 PUFA are, in general, synonymously identified to dietary LA, while seemingly ignoring other members who can contribute to tissue AA, i.e., dietary gamma-linolenic acid (GLA, 18:3 n-6) and AA.

This study was designed to explore the relationship of dietary LA and tissue AA, *viz*., phospholipid pools of plasma/serum and erythrocytes. To our knowledge, this is the first study to review the literature as to whether increasing dietary LA is positively correlated with increasing tissue AA content, and whether reducing dietary LA has the opposite effect in adults consuming Western-type diets. We further investigated what potential contributions other dietary n-6 PUFA may have on tissue AA content. This study was limited in scope and did not address other controversial issues related to dietary LA or other PUFA or their effects on issues related to health.

## Methods

**Figure 1:**
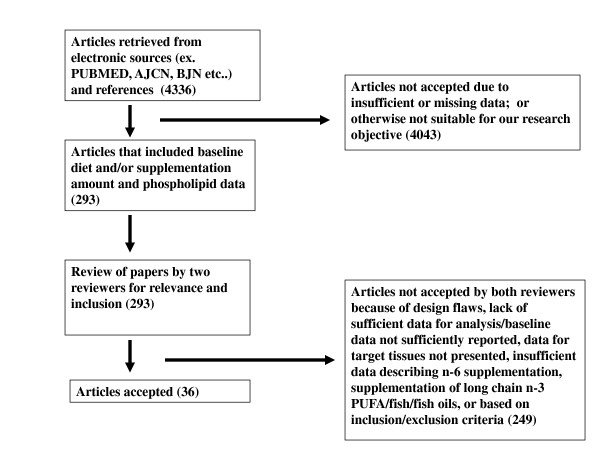
**Schematic outlining the systematic review**.

The aim of this paper was to identify, review, and evaluate all peer-reviewed published literature presenting data outlining changes in dietary LA in adult human clinical trials which report phospholipid fatty acid composition (specifically AA) in plasma/serum and erythrocytes. We chose the phospholipid pool in plasma/serum because here is where a majority of the human data is, it represents membranes of lipoproteins derived from the surface of hepatic endoplasmic reticulum [15] (it helps to control for potential variations in other components, such as circulating triglycerides). The studies reporting the fatty acid composition of erythrocyte phospholipids do so because fatty acids in erythrocytes are almost totally esterified in phospholipids. Further refinements to the search strategy included reported changes in tissue AA levels following dietary intake of AA and its various n-6 PUFA precursors, i.e., LA and GLA. Published articles meeting eligibility criteria from 1970 to present were reviewed, of which 4336 articles were retrieved from May 2009 - November 2009 (Figure 1). The primary search engine used was PubMed.gov (The National Library of Medicine, National Institutes of Health), along with several prominent nutrition-based clinical journals, i.e., American Journal of Clinical Nutrition, British Journal of Nutrition, and any additional citations in articles reviewed. The search terms included linoleic acid, γ-linolenic acid, gamma-linolenic acid, arachidonic acid, omega-6, n-6, olive oil, soybean oil, sunflower oil, safflower oil, corn oil, omega-3, n-3, plasma, erythrocyte, red blood cell and phospholipid.

The following eligibility criteria applied to all accepted articles. Subjects had to be 18 years or older with no known metabolic disorder that would influence tissue AA content. Sufficient data on LA, GLA and/or AA consumption (pre- and post-intervention) was required. The nature of the intervention (i.e., capsules, oils or dietary modifications) had to be presented. The fatty acid data (plasma/serum and/or erythrocyte) had to be determined from fasting patients, pre- and post-supplementation. Baseline and post-treatment of tissue phospholipid fatty acid composition had to be provided. On occasion, percent changes in tissue fatty acid composition were provided and this data was used. Only those papers published after 1970 due to improved gas chromatographic methods were accepted. Articles were automatically excluded if subjects were less than 18 years old, pregnant or nursing, consuming supplements containing long chain n-3 fatty acids or supplemented fish intake above and beyond their typical dietary regimen, or using known inhibitors of AA metabolism, such as non-steroidal anti-inflammatory drugs (NSAIDs).

After an initial review of the papers, 4043 were excluded because of insufficient data or studies that did not investigate our parameters. Of the 293 papers that passed the initial review process, each was reviewed by two independent investigators (BR and JW) and thirty-six were acceptable by both reviewers. Those papers that were not accepted (n = 249) were rejected because baseline data was not sufficiently reported, data for target tissues was not presented, insufficient data was present and did not allow for appropriate calculations, background diets were not sufficiently described, or they included supplementation of restricted food items (i.e., long chain n-3 PUFA). Thirty-six articles were found to meet all of the inclusion-exclusion criteria.

Once accepted, data on dietary n-6 fatty acid intake (% of calories or g/d) and tissue AA content were extracted. Study design, number and gender of subjects, method of supplementation (i.e., type of oil, capsules or food component used) were recorded. Those studies involving dietary LA as percentage of calories or g/d and its effects on changes in tissue AA content are summarized in Tables 1, 2, &3. Similarly, those studies involving dietary GLA (0.36-6.00 g/d) and AA (0.50-6.00 g/d) are summarized in Tables 4 and 5. If a study met the eligibility criteria and contained more than one comparison, each comparison was reported as a separate data point.

**Table 1:** Studies outlining the effects of decreasing dietary linoleic acid levels (% energy) from baseline on changes in plasma/serum phospholipid arachidonic acid level

Author, (reference)	Study design	Subjects	Diet length	Diet comparison	LA (% change) Baseline to intervention	Δ**AA (%) change**	Comments
Lasserre [17]	Randomized crossover	N = 24	5 months	Peanut oil diet (baseline) compared to milk fat diet	-90 (6.5%-0.6% )	9.99 N.S.	Used peanut oil group b/c close to DRI for LA. Subjects were nuns in monastery.
				Peanut oil diet (baseline) compared to low erucic acid rapeseed oil (canola oil) diet	-30 (6.5%-4.5%)	-5.49 N.S.	Used peanut oil group b/c close to DRI for LA. Subjects were nuns in monastery.
Lichtenstein [35]	Randomized double-blind crossover	N = 30	35 days	Soybean oil diet (baseline) compared to high oleic acid soybean oil diet	-82 (11%-1.9%)	-2.58 N.S.	Pooled data of men and women. Baseline diet of 10.96% energy closest to DRI for LA. AA did not differ among remaining groups tested either
Liou [37]	Randomized crossover	N = 24	4 weeks	High linoleic acid sunflower oil (diet) compared to high oleic acid safflower oil (diet)	-63 (10.5%-3.8%)	-5.55 N.S.	Incorporated test oils into baked foods (cookies, breads), mayonnaise, salad dressing. AA data presented in graphs, not tables. Fish intake was avoided for all groups. AA PL content did not differ between sequence of diets going from high LA to low LA or vice versa. Study address low or high LA with constant ALA at 1%.
Goyens [38]	Double-blind intervention	N = 19	6 weeks	Reduced LA in food items (margarines, pastries, baked goods)	-57 (7%-3%)	5.26 N.S.	Test oils consumed in margarine and pastries. Prohibited consumption of fish or marine foods in all groups.
Vega-Lopez [36]	Randomized crossover	N = 15	35 days	Canola oil diet compared to palm oil diet	-50 (6.5%-3.3%)	-8.06 N.S.	Canola oil in mixed foods was replaced by palm oil in mixed foods. AA did not change among all three dietary groups. Canola oil diet is baseline because closest to DRI LA intake.
Li [39]	Parallel intervention	N = 17	28 days	High LA diet to moderate LA diet using canola oil/canola margarine	-48 (13.5%-7%)	-3.80 N.S	Subjects were given diet more than twice DRI for LA and then given diet resembling the DRI for LA. All groups were asked to not consume fish.
				High LA diet to normal LA diet using canola oil/canola margarine	-39 (11.9%-7.3%)	-16.5 N.S.	
Mantzioris [40]	Parallel intervention	N = 15	4 weeks	Control diet (sunflower oil) group compared to intervention diet (flaxseed oil)	-57 (7.8%-3.3%)	-4.5 N.S.	Control group consumed relatively close to DRI for LA while intervention group reduced LA by more than half.
King [16]	Randomized parallel	N = 66	6 weeks	Baseline diet compared to low fat diet	-29 (10%-7.1%)	4.1 (p < 0.05)	Used modified food items for diets containing different amounts of fat. Reported AA PL in % change.
Geppert [41]	Randomized double- blind intervention	N = 54	8 weeks	Baseline diet compared to LA reduced diet (using olive oil capsules)	-12 (5.8%-5.1%)	1.12 N.S.	Used olive oil capsules with vegetarians.

Percent change (±) from baseline in AA that is significant is identified with (p < 0.05). Percent change (±) from baseline in AA that is not significant is denotedby N.S.

Abbreviations: AA, arachidonic acid; DRI, Dietary Reference Intake; LA, linoleic acid; PL, phospholipid

**Table 2:** Studies outlining the effects of increasing dietary linoleic acid levels (% energy) from baseline on changes in plasma/serum phospholipid arachidonic acid level

Author, (reference)	Study design	Subjects	Diet length	Diet comparison	LA (% change) Baseline to intervention	Δ **AA (%) change**	Comments
King [16]	Randomized parallel intervention	N = 33	6 weeks	Baseline diet compared to moderate fat diet	13 (10.3%-1.6%)	-3.2 (p < 0.05)	Used modified food items for diets containing different amounts of fat. Reported AA PL as % change.
Thijssen [42]	Randomized multiple crossover	N = 45	5 weeks	Stearic acid diet to oleic acid diet in food items (using margarines, breads, sponge cakes)	14 (2.1% -2.4%)	-2.24 (N.S.)	No order to diets given. Subjects received all 3 diets with varying amounts of LA.
				Oleic Acid to LA food items (margarines, breads, sponge cakes)	287 (2.4%-9.3%)	-1.15 (N.S.)	No order to diets given. Subjects received all 3 diets with varying amounts of LA.
Montoya [43]	Sequential interventions	N = 41	4 weeks	From palm oil based diet compared to olive oil based diet	16 (3.2%-3.7%)	4.82 (N.S.)	Used nuns and priests. Everyone consumed same sequence of diets. AA did not change among the three test diets. No crossover, subjects were their own controls.
				Olive oil based diet to sunflower oil base diet	230 (3.7%-12.2%)	-3.44 (N.S.)	
Lichtenstein [35]	Randomized double-blind crossover	N = 30	35 days	Baseline soybean oil diet compared to low saturated fat-soybean oil diet. The soybean oils varied in LA composition	15.8 (11% -12.7%)	0.89 (N.S.)	Pooled data of men and women. Five diets of random order. Subjects visited metabolic kitchen 3 times week. Varying LA amounts using modified soybean oils.
				Baseline high oleic-soybean oil diet to low ALA-soybean oil diet	552 (1.9%-2.5%)	1.27 (N.S.)	
Li [39]	Parallel intervention	N = 10 N = 7	14 days 14 days	Baseline Western diet to intervention diet increased in LA intake using safflower oil	17.8 (10.1%-11.9%)	6.18 (N.S.)	Used safflower oil/safflower margarine to increase LA in diet to almost twice DRI of LA. AA did not differ among all groups in study.
				Baseline Western diet to intervention diet increased in LA intake using safflower oil	82.4 (7.4%-13.5%)	0.96 (N.S.)	Used safflower oil/safflower margarine to increase LA in diet to more than twice DRI of LA. AA did not differ among all groups in study.
Vega-Lopez [36]	Randomized crossover	N = 15	35 days	Canola oil diet compared to soybean oil diet	64.2 (6.5%-10.7%)	-2.35 (N.S.)	Canola oil in mixed foods was replaced by soybean oil in mixed foods. AA did not change among all three dietary groups. Canola oil diet is baseline because close to the DRI for LA.
Liou [37]	Randomized crossover	N = 22	4 weeks	Low LA diet (high in oleic acid safflower oil) compared to high LA diet (high in sunflower oil)	176 (3.8%-10.5%)	-0.18 (N.S.)	AA PL content presented as graphs, not numerically. Fish was avoided in all dietary groups. ALA intake was kept constant between low/high diet.
Valsta [19]	Randomized crossover	N = 39	6 weeks	Habitual diet compared to high LA trisunflower oil diet	86 (4.2%-7.8%)	10 (p < 0.05)	Fish cut in half in all dietary groups. Has a baseline for before each diet. Used trisunflower oil in margarine, food oil, salad dressing, bread, cake and cookies, in place of habitual foods.
				Habitual diet compared to high ALA rapeseed oil diet	45 (4.2%-6.1%)	2.77 (N.S.)	
Raatz [18]	Randomized crossover	N = 10	28 days	Low fat diet (20% energy) compared to high fat diet (45% energy).	100 (6%-12%)	-16 (p < 0.05)	Modified foods rich in LA. Random order to diet, so baseline was chosen based on DRI of LA. Used washout period of 21-28 days
Lasserre [17]	Randomized crossover	N = 24	5 months	Peanut oil diet compared to sunflower oil	111 (6.5%-13.7%)	-20 (p < 0.05)	Used peanut oil group b/c close to DRI for LA.
Innis [13]	Randomized crossover	N = 24	8 weeks	Low LA diet to high LA diet	176 (3.8%-10.5%)	1.86 (N.S.)	Controlled for dietary AA.

Percent change (±) from baseline in AA that is significant is identified with (p < 0.05). Percent change (±) from baseline in AA that is not significant is denoted by N.S.

Abbreviations: ALA, alpha-linolenic acid; AA, arachidonic acid; DRI, Dietary Reference Intake; LA, linoleic acid; PL, phospholipid

**Table 3:** Studies outlining the effects of supplementing dietary linoleic acid levels (g/day) on changes in plasma/serum phospholipid arachidonic acid level

Author, (reference)	Study design	Subjects	Diet length	LA source, amount supplemented (g/d)	Δ **AA (%) change**	Comments
Anderson [44]	Parallel intervention	N = 8	3 months	Olive oil (0.2)	8.46 (N.S.)	Olive oil supplement
		N = 9		Olive oil (0.2)	9.05 (N.S.)	Provided LA for two different groups
Thies [45]	Randomized, double-blind, parallel intervention	N = 8	12 weeks	Oil blend (0.64)	3.38 (N.S.)	Different oil blends were sources of LA
				Placebo oil (0.9)	5.25 (N.S.)	
Geppert [21]	Randomized double- blind parallel intervention	N = 20	8 weeks	Oil blend (0.86)	-7 (p < 0.05)	Blend palm, rapeseed and sunflower oil
Johansson [46]	Randomized, double-blind, crossover	N = 12	4 weeks	Sea buckthorn berry oil (0.90)	-2.2 (N.S.)	Sea buckthorn berry oil is 17.9% LA.
Kew [47]	Double-blind, parallel intervention	N = 42	4 weeks	Olive oil (0.92)	-13.04 (N.S.)	
Buckley [48]	Double-blind parallel intervention	N = 45	4 weeks	Olive oil (0.95)	-7.14 (N.S.)	
Yaqoob [34]	Randomized, double- blind parallel intervention	N = 8 per group	12 weeks	Placebo (coconut/soybean oil) (1.0)	20 (N.S.)	
				Olive oil (1.2)	-2.1 (N.S.)	
				Sunflower oil (6.95)	16 (N.S.)	
Wallace [49]	Randomized, double- blind parallel intervention	N = 8	12 weeks	Oil blend (1.52)	-6.97 (N.S.)	
		N = 8		palm/soybean oil (1.7)	2.43 (N.S.)	
Miles [27]	Randomized, double-blind parallel intervention	N = 8	12 weeks	Placebo (palm/sunflower oil) (2.07)	2.19 (N.S.)	
Grimsgaard [20]	Double-blind, parallel intervention	N = 78	7weeks	Corn oil (2.24)	3.1 (p < 0.05)	
Conquer [50]	Double-blind, parallel intervention	N = 24	42 days	Corn oil (2.39)	1.12 (N.S.)	
Finnegan [51]	Double-blind, parallel intervention	N = 50	6 months	Safflower/sunflower (11.6)	7.19 (N.S.)	Test oils provided as margarine and capsules

Percent change (±) from baseline in AA that is significant is identified with (p < 0.05). Percent change (±) from baseline in AA that is not significant is denoted by N.S.

Abbreviations: AA, arachidonic acid; DRI, Dietary Reference Intake; LA, linoleic acid

**Table 4:** Studies outlining the effects of supplementing dietary gamma-linolenic acid on changes in plasma/serum phospholipid arachidonic acid level

Author, (reference)	Study design	Subjects	Diet length	GLA source, amount supplemented (g/d)	Δ **AA (%) change**	Comments
Ebden [52]	Double-blind intervention	N = 6	8 weeks	Efamol oil (0.36)	6.1 (N.S.)	No crossover with placebo. Subjects were asthmatics used medication or bronchodilator.
Thavonen [29]	Randomized, double-blind crossover	N = 15	3 weeks	Black current seed oil (0.38)	3.7 (N.S.)	Subjects aged 55-75 years old
Theis [45]	Double-blind parallel interventions	N = 8	12 weeks	GLA-rich triacylglycerol capsules (0.77)	27 (p < 0.05)	Subjects consumed capsules for 12 weeks. AA changed only on 12^th ^week.
Yaqoob [34]	Double-blind parallel intervention	N = 8	12 weeks	Evening primrose oil (1.06)	14 (N.S.)	
Mills [28]	Randomized double-blind parallel intervention	N = 10	28 days	Borage oil (1.30)	12 (p < 0.05)	AA data available for only pre and post intervention (28 days)
Miles [27]	Randomized double-blind intervention	N = 8-12	12 weeks	Borage oil capsules (2.00)	15 (p < 0.05)	Consumed capsules for 12 weeks. AA only increased after the 8th week, no difference after 8^th ^week.
Johnson [53]	Pre-post intervention	N = 5	3 weeks	Ultra-GLA capsules (6.00)	31 (p < 0.05)	

Percent change (±) from baseline in AA that is significant is identified with (p < 0.05). Percent change (±) from baseline in AA that is not significant is denoted by N.S.

Abbreviations: AA, arachidonic acid; GLA, gamma-linolenic acid

**Table 5:** Studies outlining the effects of supplementing dietary arachidonic acid on changes in plasma/serum phospholipid arachidonic acid level

Author, (reference)	Study design	Subjects	Diet length	AA source, amount supplemented (g/d)	Δ **AA (%) change**	Comments
Sinclair [54]	Parallel intervention	N = 4	7 days	White meat/eggs (0.50)	52 (p < 0.05)	Consumed AA rich, low fat diet
Ishikura [55]	Double-blind crossover	N = 25	1 month	SUNTGA40S capsules (0.72)	27 (p < 0.05)	Derived from Mortierella alpina
Theis [45]	Randomized, double-blind, parallel intervention	N = 48	12 weeks	ARASCO (0.68)	85 (p < 0.05)	Derived from Mortierella alpina
Kusmoto [56]	Double-blind intervention	N = 12	4 weeks	SUNTGA40S (0.84)	45 (p < 0.05)	Derived from Mortierella alpina
Nelson [57]	Single blind crossover intervention	N = 10	50 days	ARASCO (1.49)	85 (p < 0.05)	Had 65 day washout period. Derived from Mortierella alpina
Seyberth [58]	Single blind intervention	N = 4	2-3 weeks, depending upon subject	Capsules, AA ethyl ester (6.00)	136 (p < 0.05)	Averaged from all 4 subjects

Percent change (±) from baseline in AA that is significant is identified with (p < 0.05). Percent change (±) from baseline in AA that is not significant is denoted by N.S.

Abbreviations: AA, arachidonic acid

Baseline tissue AA levels were defined as relative abundance of AA in tissue phospholipids prior to dietary supplementation (or reduction) of the corresponding dietary n-6 PUFA of interest (i.e., LA, GLA or AA). In the case of cross-over designs, baseline fatty acid composition was established following a washout period or after supplementation of a control diet if there were no or only minor changes in the dietary n-6 PUFA content. For example, a supplement rich in oleic acid (a monounsaturated fatty acid typically used as a control and known to have a neutral effect on tissue AA content) could be used as a control lipid (or oil) prior to supplementation of an equal amount of a lipid (or oil) rich in LA. Percent change for each dietary n-6 fatty acid of interest was used to standardize the relative differences between baseline intakes and intervention intakes following the experimental period using the following formula:

The levels of intake were based on the relative caloric amount (% of calories), and when this data was not available absolute intake levels (g/d) were used. Percent change for tissue AA content was used to standardize the relative differences between baseline levels and intervention levels following the experimental period using the following formula:

### Statistical Analysis

The overall linear correlation between percent change of dietary n-6 fatty acids and percent change of tissue AA was computed using the Proc Corr procedure in SAS 9.2 (SAS Institute Inc. SAS Campus Drive, Cary, North Carolina). The correlation matrix and the T statistic tested for correlation and statistical significance, respectively. For the linear correlations, the equation of the line was computed, and represented by y = mx for those that exhibited linearity. Data not resembling a linear relationship (i.e. dietary GLA and AA) utilized a polynomial growth curve from SAS General Linear Model and t-tests for model parameters tested for significance. P-values less than or equal to 0.05 were considered significant. The Y values represent changes of AA (% from baseline) and the X values represent the changes of the various dietary n-6 PUFA (% from baseline or g/d supplemented). In addition to the overall correlation test, the statistical significance for each individual data point (for changes in tissue AA), as reported by the authors in their respective manuscripts, was identified in each graph. If the changes from baseline were significantly different the data was represented by triangle. If the changes from baseline were not statistically different, they were represented by a diamond.

## Results

**Figure 2:**
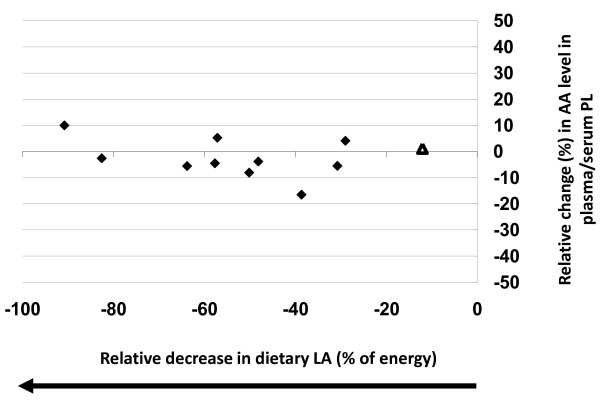
**Effects of decreasing dietary linoleic acid (LA) intake (% change) based on energy on changes in plasma/serum phospholipid arachidonic acid (AA) content**. Significant changes (p < 0.05) in AA as reported in the original papers are designated as triangles. Non-significant AA changes as reported in the original papers are designated as diamonds. Abbreviations: AA, arachidonic acid; LA, linoleic acid; PL, phospholipid.

Eleven comparisons reported decreases in LA intakes (-12% to -90%) and no significant correlations were associated with changes in plasma/serum phospholipid AA content (r^2 ^= 0.07, p = 0.44, y = 0.026x) (Table 1 and Figure 2). Only one study of the eleven reported a significant change, a 4.1% increase in AA content, following a 29% reduction in LA intake [16].

**Figure 3:**
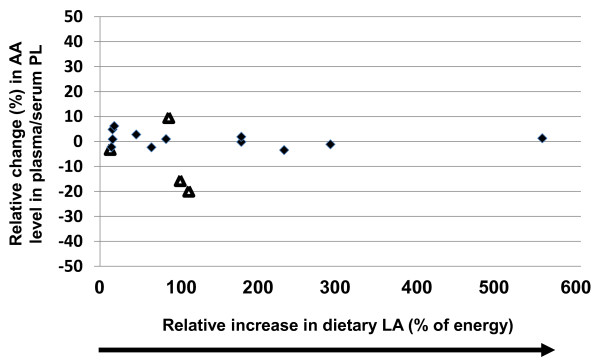
**Effects of increasing dietary linoleic acid (LA) intake (% change) based on energy on changes in plasma/serum phospholipid arachidonic acid (AA) content**. Significant changes (p < 0.05) in AA as reported in the original papers are designated as triangles. Non-significant AA changes as reported in the original papers are designated as diamonds. Abbreviations: AA, arachidonic acid; LA, linoleic acid; PL, phospholipid.

**Figure 4:**
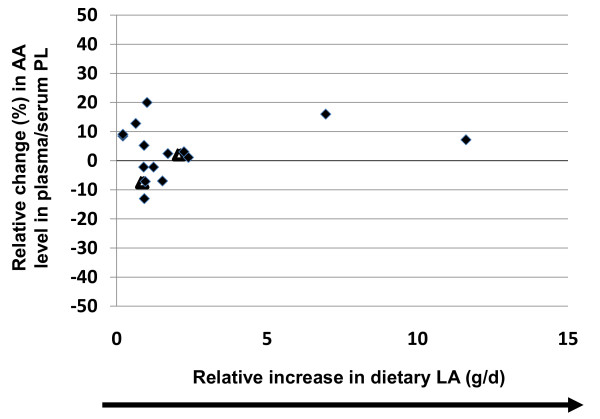
**Effects of increasing dietary linoleic acid (LA) intake (g/d) on changes in plasma/serum phospholipid arachidonic acid (AA) content**. Significant changes (p < 0.05) in AA as reported in the original papers are designated as triangles. Non-significant AA changes as reported in the original papers are designated as diamonds. Abbreviations: AA, arachidonic acid; LA, linoleic acid; PL, phospholipid.

Increases in dietary LA, ranging from 12%-550%, was not significantly correlated with changes in plasma/serum phospholipid AA content (r^2 ^= 0.074, p = 0.45, y = -0.0053x) (Table 2 and Figure 3). Of the sixteen comparisons, only four studies reported significant changes in AA levels when dietary LA levels were increased; three studies reported 3-20% reductions following 12%-110% increases in LA consumption [16-18] and only one study reported a significant increase in AA content (10%) following an 86% increase in LA intake [19]. Sub-dividing the studies by design (crossover versus non-crossover) had no effect on the results (data not shown). Similarly, in those studies that only reported absolute levels of LA supplementation (g/d), increasing LA supplementation was not significantly correlated with changes in plasma/serum phospholipid AA content (r^2 ^= 0.092, p = 0.64, y = 0.969x) (Table 3 and Figure 4). Of the seventeen comparisons, only two were significantly different, one resulted in an increase in AA content by 3% following supplementation of 2.24 g/d of LA [20] and the other resulted in a reduction of AA content by 7% following supplementation of 0.86 g/d [21].

**Figure 5:**
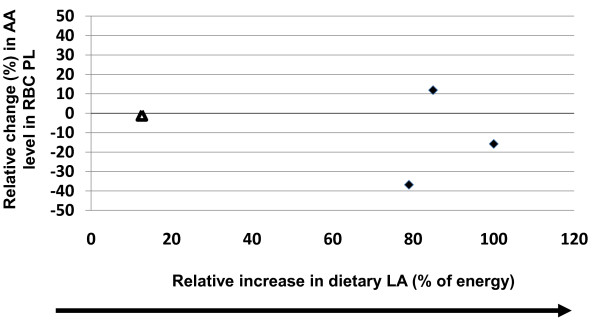
**Effects of increasing dietary linoleic acid (LA) (% change) intake based on energy on changes in erythrocyte (RBC) phospholipid arachidonic acid (AA) content**. Significant changes (p < 0.05) in AA as reported in the original papers are designated as triangles. Non-significant AA changes as reported in the original papers are designated as diamonds. Abbreviations: AA, arachidonic acid; LA, linoleic acid; PL, phospholipid.

**Figure 6:**
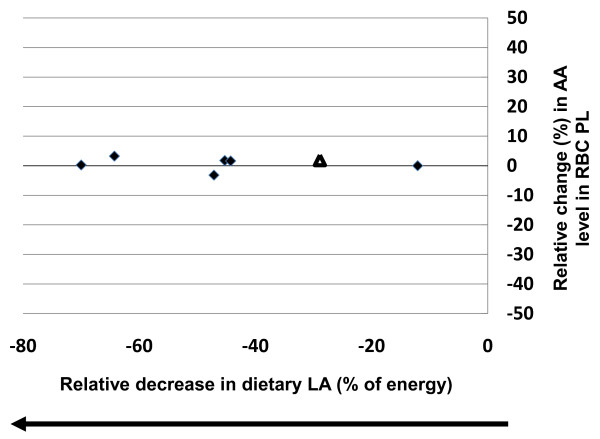
**Effects of decreasing dietary linoleic acid (LA) (% change) based on energy on changes in erythrocyte (RBC) phospholipid arachidonic acid (AA) content**. Significant changes (p < 0.05) in AA as reported in the original papers are designated as triangles. Non-significant AA changes as reported in the original papers are designated as diamonds. Abbreviations: AA, arachidonic acid; LA, linoleic acid; PL, phospholipid.

Similar comparisons were made in erythrocytes with increasing and decreasing intakes of LA, although the number of studies were more limited. Increases in dietary LA, ranging from 12%-100%, were not significantly correlated with changes in tissue AA content (r^2 ^= 0.06, p = 0.75, y = -0.1479x) (Figure 5). Reducing dietary LA intake (-12% to -70%) was not significantly correlated with changes in tissue AA content (r^2 ^= 0.017, p = 0.77, y = -0.0174x) (Figure 6). In addition, out of the seven studies, only one study reported a significant change where decreasing dietary LA intake by 29% resulted in a 4% increase in AA content [16].

**Figure 7:**
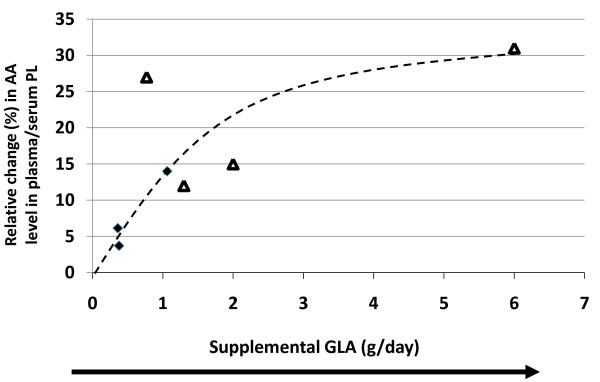
**Effects of increasing dietary gamma-linolenic acid (GLA) (g/d) on changes in plasma/serum phospholipid arachidonic acid (AA) content**. Significant changes (p < 0.05) in AA as reported in the original papers are designated as triangles. Non-significant AA changes as reported in the original papers are designated as diamonds. Abbreviations: AA, arachidonic acid; GLA, gamma-linolenic acid; PL, phospholipid.

Seven studies met our criteria for the effects of GLA supplementation on changes in plasma/serum phospholipid AA content (Table 4 and Figure 7). There appeared to be a dose dependent increase in AA content with increasing intakes of GLA (ranging from 0.36 g/d to 6.00 g/day). This positive correlation (r^2 ^= 0.75, p = 0.03, y = 0.004x + 7.36) was significant with a linear regression model, and approached significance with a non-linear (quadratic) regression model (r^2 ^= 0.56, y = -1.4x^2 ^+ 13.76x, p = 0.079). The number of available data may be a factor in these results. Of the six GLA data points, four of them reported statistically significant increases in AA.

**Figure 8:**
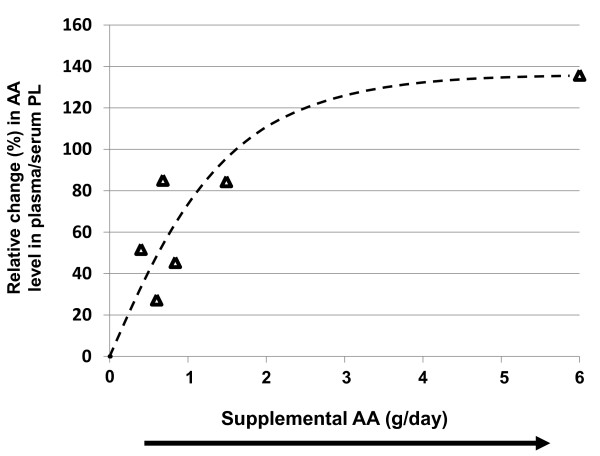
**Effects of increasing dietary arachidonic acid (AA) (g/d) based on energy on changes in plasma/serum phospholipid AA content**. Significant changes (p < 0.05) in AA as reported in the original papers are designated as triangles. Non-significant AA changes as reported in the original papers are designated as diamonds. Abbreviations: AA, arachidonic acid; PL, phospholipid.

Similiarly, increasing dietary AA (0.50 g/d to 6.00 g/day) was positively correlated with increases in plasma/serum phospholipid AA content using a quadratic regression model (r^2 ^= 0.79, y = -8.7x^2 ^+ 74.87x, p = 0.013) (Figure 8). All data points were reported as significantly different (Table 5).

## Discussion

Arachidonic acid is arguably the most important PUFA associated with membrane phospholipids. Upon release, AA can be enzymatically metabolized to a myriad of bioactive derivatives, eicosanoids, known to contribute to a variety of chronic diseases, but are also known to be involved in tissue homeostasis and the resolution of inflammation [1-4,22]. The relative abundance of AA in membrane phospholipids positively influences eicosanoid production [23]. It is well known that dietary PUFA can affect tissue AA levels; however, what is uncertain and controversial is whether modifying current intakes of dietary LA will result in concomitant changes in tissue AA content, i.e., increasing LA intake results in an increase in tissue AA content and decreasing LA has the opposite effect [8]. The goal of this paper was to ascertain the relationship between dietary LA and tissue AA content (phospholipid pools of plasma/serum and erythrocytes) in adults consuming a Western-style background diet. It was not designed to address other controversies surrounding the issues of dietary n-6 or n-3 PUFA or in other population groups.

Many papers interchange the more general term n-6 PUFA for dietary LA, but there are two major n-6 PUFA, LA and AA, that are distributed unevenly in the Western diet. While LA is the major PUFA in most commonly consumed foods, AA is exclusively found in animal products, such as, muscle, organ meats and eggs [24]. They have distinct biological activities that are biochemically linked via desatuation and elongation, and as such, LA is the conditionally essential fatty acid. Linoleic acid is specifically required in the skin to maintain the integrity of the epidermal water barrier and AA is the immediate precursor to eicosanoids, as well as being the n-6 PUFA selectively incorporated into the membranes of certain tissues, i.e., brain [25]. When consumed (LA vs. AA), they appear to have differential effects on tissue fatty acid composition, where AA appears to more robustly modify tissue AA levels and eicosanoids [14,26].

The data presented in this paper suggests that a dose response between dietary LA and tissue AA does not exists within the backdrop of individuals consuming a Western-type diet. Increasing LA by as much as 551% from baseline and reducing LA by as much as 90% from baseline failed to yield compelling evidence supporting the concept that any conversion of dietary LA to downstream metabolites results in tissue enrichment of AA, a notion commonly assumed. For example, *"However, the higher concentrations of LA typically found in the Western diet results in a greater conversion of LA to arachidonic acid" *[8] and*"Excessive n-6 precursors promotes formation of AA" *[9], suggesting enrichment of AA in tissues with increases in LA intake. We chose to evaluate the data by looking at changes from baseline in tissue AA content to standardize the data from one study to the next. Each study began with a baseline value and we reported percent changes from that baseline. Supplemental intakes of LA were reported based on energy and when that value could not be determined, we reported absolute supplemented values, and these data were reported seperately.

As observed from the distribution of the responses, there was wide variability. Some papers showed small increases in tissue AA levels when dietary LA changed, while other papers showed small decreases, but most of these changes lacked significance. When there was significance, the changes were minimal and the distribution pattern of the data did not favor an increase or a decrease. We chose plasma/serum and erythrocytes as the tissues of choice because here is where the bulk of data exists in the human literature. Erythrocytes represent a more stable pool of dietary lipids, contain very little neutral lipids and thus represents a membrane fraction of AA. Fasting plasma/serum phospholipid levels primarily (but not exclusively) represents in part phospholipids of lipoproteins that are derived from hepatic endoplasmic reticulum [15], and this pool is more responsive to more recent dietary PUFA intakes.

In an effort to identify why dietary LA may not modify tissue AA levels, we reviewed the literature for dietary GLA using the same search strategy. Was the conversion of LA to AA rate-limiting, or were tissue levels of AA saturated? Delta-6 desaturase is the rate-limiting enzyme in the metabolism of LA to AA. GLA is a dietary n-6 PUFA that enters the metabolic pathway after the delta-6 desaturase step. If delta-6 desaturase is rate-limiting and tissue AA content is not saturated, then there should be evidence that including GLA in the diet increases tissue AA levels. When GLA was supplemented as the triacylglycerol form or as a component of a dietary oil containing GLA (i.e., blackcurrant, evening primrose or borage oil), tissue AA content increased in a dose responsive manner. These effects appeared to be less prominent in those studies [27-29] that used oils containing appreciable amounts of the more highly unsaturated n-3 PUFA stearidonic acid, i.e., blackcurrant [30]. When AA was supplemented in the diet, there was further enrichment in tissue AA content above that observed with either LA or GLA. These results suggest that delta-5 desaturase potentially becomes rate limiting when GLA is supplemented. The reaction mediated by delta-5 desaturase is an intermediate step between GLA and AA and by-passing that step with dietary AA leads to further enrichment. These data seem to suggest that while dietary LA maybe a metabolic precursor for AA, its influence on tissue levels in populations consuming Western diets are limited by the enzymatic conversion through delta-6 desaturase and not due to tissue saturation of AA. These data are supported by the poor rates of conversion of plasma/serum LA to AA in adults. In tracer studies involving stable isotopes, the estimated fractional conversion of LA to AA was between 0.3% and 0.6% [31].

The levels of LA in the diet required to achieve essentiality could be as low as 0.5-2.0% of energy in infants [32,33] and it has been reported that tissue levels of AA no longer respond to dietary LA intakes above 2% energy in adults [12]. Our study was designed to chose studies that incorporated a Western-type diet where LA is not typically limiting, reflective of the general public. This means a full compliment of PUFAs were being consumed along with LA supplementation. The DRIs for LA and alpha-linolenic acid (ALA, 18:3 n-3) are 12 g-17 g/d and 1.1 g-1.6 g, respectively (women the lower figure, men the higher figure). This would be equivalent to intakes approximating 6% and 0.7% of calories per day for LA and ALA, respectively. It is not unreasonable to think that with a background diet containing LA, ALA, AA, and long-chain n-3 PUFAs, i.e. eicosapentaenoic acid (EPA, 20:5 n-3) and docosahexaenoic acid (DHA, 22:6 n-3) at typical intakes, that modifying dietary LA levels may not influence tissue AA levels. It is possible that as LA increases in the diet it maybe competing with AA for reacylation into phospholipids [13,14,16-18,21,34].

A small number of studies modified LA intakes by using oils that also contained some ALA, such as soybean and canola oil [17,35,36], but the results from these studies were not significant and were similar to the other results. There could be some concern that some of the supplemented oils contain ALA, such as soybean oil. It must be remembered that soybean oil has a LA:ALA ratio similar (8:1) to that found in the US diet (10:1) and if you included or excluded these papers the results were unaffected. We also included two studies that supplemented LA with recommended fish restrictions (because they met our inclusion/exclusion criteria) [19,37]. One study (+176% LA) reported no changes in AA levels, while the other (+86% LA) reported a 10% increase in AA.

Some of the weaknesses of this review are reflected in the studies that qualified for our evaluation. Most were not designed to specifically address our research question; however, those that were specifically designed to evaluate the effect of dietary LA on tissue AA content yielded results that were similar to the overall results [13]. Each study used a different population with potentially different background diets, but overall this would better reflect the consumption patterns of the general public. Not all studies were blinded (61% were blinded) and dietary LA was not exclusively modified. The methods for modifying LA intakes were varied and other dietary PUFA were not controlled for with the exceptions identified previously, and data for only two tissues were evaluated. When LA was modified, it was done so by typically changing the levels of an oil rich in LA (i.e., corn oil, safflower oil, sunflower oil) or foods containing LA (as opposed to adding pure LA), reflecting how LA would be consumed by the general public. There were no standard length to the studies. For example, studies involving plasma/serum ranged between 14 days-5 months, and those looking at erythrocyte data ranged between 14-180 days. Importantly, the subjects were used as their own controls, the studies addressed changes in LA in relationship to Western-type diets, and the results were not different between those studies that were double-blind randomized placebo controlled trials (1/3) and those that were not. Despite these weaknesses, positive results were still identified with intakes of GLA and AA, helping to support those results reported with LA.

## Conclusions

Elevated tissue AA levels are believed to be positively associated with eicosanoid formation and risk for a variety of chronic diseases, including cardiovascular disease, cancer and inflammation. The literature expresses concern over the fact that increasing dietary LA can potentially enrich tissues with AA due to their metabolic link. The results of this study do not support this concern. Whereas AA levels in blood phospholipids is increased by GLA or AA supplements, intervention studies bring no evidence to suggest that changes in dietary LA will modify tissue AA content in an adult population consuming a Western-type diet.

## Abbreviations

AA: arachidonic acid; ALA: alpha-linolenic acid; DHA: docosahexaenoic acid; DRI: Dietary Reference Intake; EPA: eicosapentaenoic acid; GLA: gamma-linolenic acid; LA: linoleic acid; NSAIDs: non-steroidal anti-inflammatory drugs; PL: phospholipid; PUFA: polyunsaturated fatty acids.

## Competing interests

The authors declare that they have no competing interests.

## Authors' contributions

BR conducted the research and co-wrote manuscript, and JW formulated and designed research, co-wrote manuscript and had final responsibility for all parts of the manuscript. All authors have read and approved the final manuscript.
